# Depression after Stroke and Risk of Mortality: A Systematic Review and Meta-Analysis

**DOI:** 10.1155/2013/862978

**Published:** 2013-03-07

**Authors:** Francesco Bartoli, Nicoletta Lillia, Annamaria Lax, Cristina Crocamo, Vittorio Mantero, Giuseppe Carrà, Elio Agostoni, Massimo Clerici

**Affiliations:** ^1^Department of Surgery and Interdisciplinary Medicine, University of Milano-Bicocca, 20900 Monza, Italy; ^2^Stroke Unit, Neurology Department, Azienda Ospedaliera Niguarda Cà Granda, 20162 Milan, Italy; ^3^Department of Mental Health, Azienda Ospedaliera San Gerardo, 20900 Monza, Italy; ^4^Department of Mental Health Sciences, University College London, London W1W 7EJ, UK

## Abstract

*Background.* Depression after stroke may have great burden on the likelihood of functional recovery and long-term outcomes. *Objective*. To estimate the association between depression after stroke and subsequent mortality. *Methods*. A systematic search of articles using PubMed and Web of Science databases was performed. Odds ratios (ORs) and hazard ratios (HRs) were used as association measures for pooled analyses, based on random-effects models. *Results*. Thirteen studies, involving 59,598 subjects suffering from stroke (6,052 with and 53,546 without depression), had data suitable for meta-analysis. The pooled OR for mortality at followup in people suffering from depression after stroke was 1.22 (1.02–1.47). Subgroups analyses highlighted that only studies with medium-term followup (2–5 years) showed a statistically significant association between depression and risk of death. Four studies had data suitable for further analysis of pooled HR. The meta-analysis revealed a HR for mortality of 1.52 (1.02–2.26) among people with depression after stroke. *Conclusions*. Despite some limitations, this paper confirms the potential role of depression on post stroke mortality. The relationship between depression and mortality after stroke seems to be related to the followup duration. Further research is needed to clarify the nature of the association between depression after stroke and mortality.

## 1. Introduction

Stroke represents the third most common cause of death in developed countries, following only coronary heart diseases and cancer [1]. It is frequently associated with higher risk for a wide range of physical and neuropsychological consequences [2, 3]. Although the importance of poststroke psychiatric comorbidity is currently well documented, it had been previously underestimated [4]. In the 1970s, the identification of mood disorders, especially depression, as specific complications following stroke introduced the concept that clinical depression after stroke could be an organic consequence of the brain damage rather than an understandable psychological reaction to motor disability [5, 6]. Since then, research on depression after stroke has gained momentum [7].

However, despite the large bulk of the literature which has been published on this topic, there is still uncertainty in relation to depression after stroke prevalence, etiology, and management. Although the risk of all depressive disorders was reported ranging from 25% to 79% among people suffering from a stroke [21], poststroke major depression prevalence ranged from 3% to 40% [22]. Data available from 51 studies that have been run between 1977 and 2002 confirmed that depressive symptoms were assessed in 33% (29–36%) among all stroke survivors at any time during followup [23]. Similar estimates were described by more recent studies [24, 25]. Variations in depression after stroke prevalence rates across studies seem to arise from differences in criteria that are used to define depression, stroke and patients' characteristics and timing of mood detection, as well as the complexity in recognition, assessment, and diagnosis of this disorder in post-stroke settings [23]. Risk of inappropriate diagnosis is high [26, 27] because of the difficulties in the assessment of mood abnormalities in patients with neurological deficits, particularly associated with dysphasia and dementia, who often experience many concurrent and “overlapping” somatic, cognitive, and affective symptoms [28]. 

However, early screening and diagnosis of depressed mood might be relevant, since depression after stroke is related to poorer outcomes. Based on main available evidence, together with worse long term functional outcomes [29], depression after stroke is associated with reduction in rehabilitation treatment efficacy [30], limitations in daily living activities [29, 31], cognitive impairment [32, 33], and a higher risk of recurrent stroke [34]. Furthermore, depression after stroke was reported to be related to a high mortality risk [35]. Some reviews examined prevalence rates and clinical correlates of depression [7, 23, 36], but data on the impact of depression after stroke on survival need to be clarified and systematically analyzed. Therefore, we performed a systematic review and meta-analysis in order to explore the relationship between depression and subsequent mortality in the poststroke population.

## 2. Methods

The present paper was conducted according to the Meta-Analyses of Observational Studies in Epidemiology (MOOSE) guidelines [37].

### 2.1. Search Strategy

We used PubMed (date range: January, 1, 1990 to November, 25, 2012) and Web of Science (date range: January, 1, 2002 to November, 25, 2012) electronic databases for search purposes. No restrictions of language were set. We used the following terms for the PubMed search strategy: (1) “Depression” [Mesh]; (2) “depression” [all fields]; (3) “Stroke” [Mesh]; (4) “post-stroke” [title/abstract]; (5) “post stroke” [title/abstract]; (6) “Mortality” [Mesh]; (7) mortal* [title/abstract]; and (8) death* [title/abstract]. We combined the terms as follows: (1 or 2) and (3 or 4 or 5) and (6 or 7 or 8). A similar search strategy was used for Web of Science database, combining the following topic terms: (1) depress*; (2) mood*; (3) affective*; (4) stroke*; (5) post-stroke*; (6) poststroke*; (7) mortal*; and (8) death*. The search phrase was built as follows: (1 or 2 or 3) and (4 or 5 or 6) and (7 or 8). 

### 2.2. Eligibility Criteria

We included studies with the following characteristics:

- assessment of depression in a sample of people suffering from a previous stroke;
- estimation of the association between depression after stroke at baseline and subsequent mortality at followup;
- additional available data on mortality also in a comparison group suffering from stroke but without depression.

Exclusion criteria were based on the following:

- depression diagnosed exclusively before stroke admission;
- results shown as continuous or quantitative scores based on psychometric scales without any dichotomization around a standardized cut-off value for depression;
- data replicated in multiple works whose inclusion would involve duplication of data.

### 2.3. Data Collection Process

A preliminary screening (reading of titles and, if needed, of abstracts) was performed in order to include all potentially relevant articles. After the first screening, the final eligibility was assessed retrieving papers in full text. Two investigators (F. Bartoli and N. Lillia) independently performed both first and final screenings of papers. When differences of opinion between reviewers occurred, these were resolved by discussion with a third member (A. Lax) of the research team, and consensus was thereby reached.

### 2.4. Data Extraction

We developed a specific data extraction sheet. One author (F. Bartoli) extracted data from the included studies and another (C. Crocamo) checked the accuracy for the inclusion in statistical software. Any disagreement was resolved by discussion with the other authors. We extracted the following information from each included study: year of publication, country, study design, sample size, depression definitions and measures, duration of followup, reported association measure (e.g., hazard ratio, relative risk), main results. When there was any uncertainty about the data, we contacted the corresponding author for clarification. We collected also information suitable for a basic quality evaluation of studies included, based on the comparability between exposed and nonexposed groups, the risk of selection bias, the evaluation of representativeness of recruited samples, and the reliability of depression assessment. 

### 2.5. Data Analysis

We analyzed data using the Review Manager (RevMan) 5.1 software [38] and STATA statistical software package, version 10 [39]. For articles providing both major and minor depression data, we analyzed only specific data of subgroups with major depression, excluding patients with minor depression. For studies showing results at different followup periods, we included just the results at the longer followup. We performed two different pooled analyses based on two different association measures, odds ratio (OR) and hazard ratio (HR) with related 95% confidence intervals, according to the available data from the included papers. The HR is commonly used in the medical literature when describing survival data, and it is defined as the estimate of the ratio of the probability that if the analyzed event has not already occurred, it will occur in the next time interval, divided by the length of that interval in the index group versus the control group. HR is the risk at any instance of followup, whereas OR quantifies the association at the end of followup. For the HRs, we estimated log hazard ratios and standard errors obtained from Cox proportional hazards regression models. Results were summarized using conventional forest plots. Random-effects models for estimating pooled effects were considered preferable rather than fixed-effect models because high variability across the included studies was expected, for example, in relation to followup duration, recruitment inclusion/exclusion criteria, setting, and depression after stroke definition. We performed subgroups analysis based on the followup duration of the included studies. The results are structured by three followup periods, short term (<2 years), medium term (2–5 years), and long term (>5 years). We performed sensitivity analyses excluding studies with potential methodological issues based on the low representativeness of recruited samples or on the lack of reliability of methods assessing depression. The presence and the level of heterogeneity were assessed using *Q* test and *I* ^2^ statistic, respectively. A funnel plot was created in order to visually inspect the risk of publication bias. We performed Egger's test for the statistical estimation of publication bias.

## 3. Results

### 3.1. Study Selection

**Figure 1:**
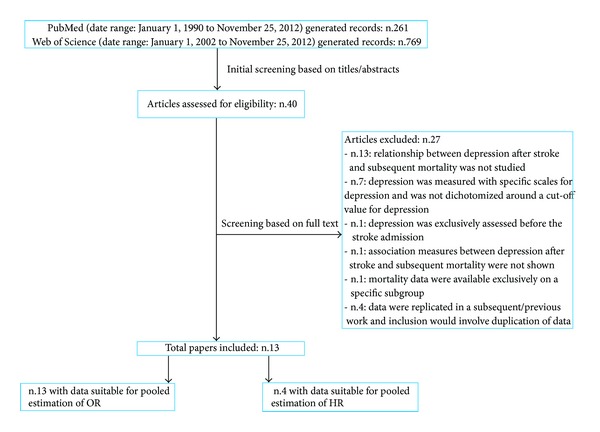
Flowchart: search results and excluded/included studies.

261 and 769 records were generated from PubMed and Web of Science databases, respectively. The preliminary screening based on titles and, where needed, abstracts identified 40 papers as potentially relevant. These papers were retrieved in full text. Among these, 27 were excluded because they did not meet the inclusion criteria. Detailed reasons for ineligibility are shown in flow diagram (Figure 1). 13 studies [8–20] were included for meta-analysis, all had data suitable for pooled estimation of the OR and four for pooled analysis of the HR mortality. 

### 3.2. Study Characteristics

All articles were in English. The year of publication ranged from 1993 to 2011. Seven papers were from USA, two from Australia, and four from Europe. There is a high variability among studies in terms of study design, sample size, male/female ratio, methods and time of detection of depression, and followup length. The followup period ranged between 12 months and 10 years. Detailed characteristics of the included papers are described in Table 1.

### 3.3. Odds Ratio of Mortality among People with Depression after Stroke

**Figure 2:**
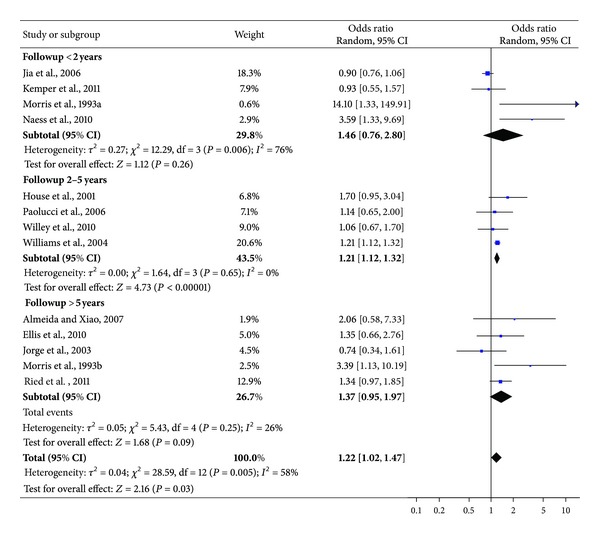
OR of mortality among subjects with depression after stroke.

The studies (n.13) involved 59,598 subjects: 6,052 with depression after stroke and 53,546 from comparison groups. 1,544 cases of death at followup were detected from depression after stroke sample and 18,216 from people without depression. The pooled OR (95% CI) for mortality at followup in people with depression was 1.22 (1.02–1.47) (Figure 2). Heterogeneity was high (*χ* ^2^ = 28.59; *P* = 0.005; *I* ^2^ = 58%). According to subgroups analyses, a statistically significant association between depression after stroke and mortality was found exclusively for studies with medium term followup. However, this result was influenced by the large size study by Williams and colleagues [20] that accounts for most of the weight of the overall effect. 

**Figure 3:**
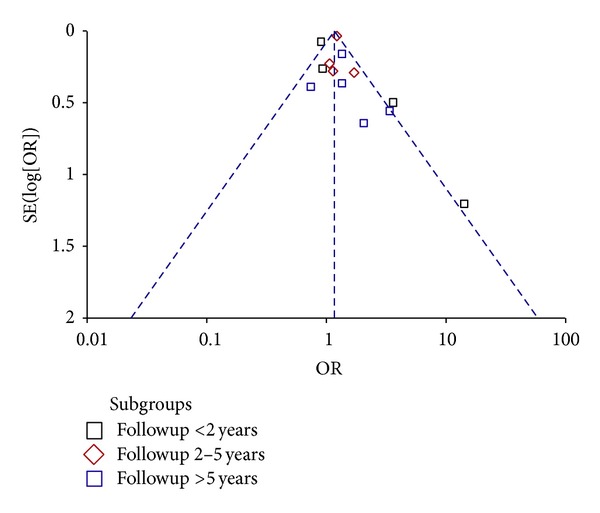
Funnel Plot. Egger's test: bias = 0.68 (−0.53–1.90); *P* = 0.241.

Funnel plot for publication bias is shown in Figure 3. Egger's test was not statistically significant (coefficients = 0.68 (−0.53–1.90); *P* = 0.241).

### 3.4. Hazard Ratio of Mortality among People with Depression after Stroke

**Figure 4:**
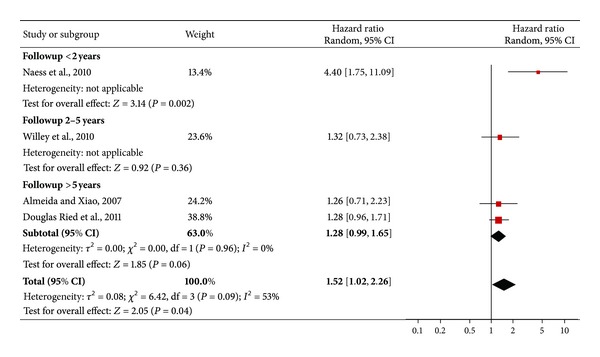
HR of mortality among subjects with depression after stroke.

The pooled HR (95% CI) for mortality at followup in people with depression after stroke was 1.52 (1.02–2.26) (Figure 4). Test for heterogeneity showed an *I* ^2^ = 53% (*χ* ^2^ = 6.42; *P* = 0.09). 

### 3.5. Quality Assessment and Sensitivity Analyses

There was no risk of poor comparability between depressed and not depressed subjects, since all studies recruited both exposed and nonexposed cohorts from the same sources. However, most of the included papers were prone to some methodological issues and potential risk of bias. In one study [9], the presence or the absence of a history of stroke in the selected population was ascertained on the basis of self-report information rather than on clinical diagnosis or medical records, introducing a potential risk of selection bias. Furthermore, data from two studies [10, 12] were based on participants of clinical trials, who, unlike those derived from purely observational studies, may not be equally representatives of the reference population. The difference between individuals allocated for receiving a specific treatment and individuals treated with placebo or other active controls might actually introduce a potential performance bias. Finally, three studies [11, 18, 20] investigated the effect of depression after stroke in special populations (veterans), not representatives of general population suffering from stroke. The sensitivity analysis excluding the studies based on samples of veterans [11, 18, 20], individuals recruited from clinical trials [10, 12], and self-report stroke patients [9] highlighted a pooled OR of 1.61 (1.01–2.55).

As regards the methods to assess depression after stroke, we found several quality issues. Five studies [8, 11, 13, 18, 20] collected relevant information from clinical records and/or administrative data. These sources may have lower sensitivity than rating scales or structured clinical interviews, since the risk of an inadequate diagnosis of depression after stroke in clinical practice is often high [40]. Furthermore, eight studies [9, 10, 12, 14–17, 19] used psychometric scales (e.g., Hospital Anxiety and Depression Scale; Hamilton Rating Scale for Depression) to evaluate depressive symptoms. Among these, four studies [10, 12, 14, 15] combined the psychometric evaluation with structured (Composite International Diagnostic Interview) or semistructured (Present State Examination) interviews to assess depression. However, the specific risk of developing the mortality outcome among individuals suffering from major depression, and not from other depressive disorders (e.g., “minor” depression or dysthymia), was available from just three studies (two out of these were based on the DSM-III criteria) [10, 14, 15]. The sensitivity analysis including only these studies showed a pooled OR of 2.75 (1.14–6.65).

## 4. Discussion

### 4.1. Main Findings

This paper on the mortality risk in subjects suffering from depression after stroke identified 13 studies with data suitable for meta-analysis. There are three main findings. First, suffering from depression after stroke has an important bearing on the chances of death, with an OR of 1.22 (1.02–1.47) (95% CI). The sensitivity analyses based on specific characteristics of recruited samples and on the methods to detect depression confirm the statistical significance of the relationship between baseline depression and risk of subsequent mortality among people with stroke. 

Second, the relationship between depression and mortality seems to be related to the duration of observation. Subgroup analysis of short term studies (<2 years) did not show a statistically significant association between depression after stroke and mortality, whereas subgroup analysis of long term studies (>5 years) showed some trend. On the other hand, subgroup analysis of medium term studies (range: 2–5 years) showed results above the threshold of statistical significance. It should be noted that the studies with followups longer than 5 years, actually considered really long intervals, ranging from 7 to 10 years. Therefore, we can assume that the burden of depression on the risk of mortality among stroke patients emerges only after 2–5 years from the index stroke, whereas, afterwards, this difference may be mitigated by the aging of stroke survivors. 

However, we need to point out that there are some alternative explanations to this result. Long term followup subgroup included studies for an overall size of 362 patients suffering from depression, so the lack of a statistical significant association may be due to the small sample sizes of the included papers. Furthermore, the study of Jorge and colleagues [12], a placebo-controlled trial of antidepressants with prospective design, represents an outlier within the long term followup subgroup (OR: 0.74 (0.34–1.61)) that may have consistently influenced the pooled OR in this subgroup. Lastly, we need to highlight that the study of Williams and colleagues [20] on a National Cohort of Veterans hospitalized following an ischemic stroke accounted for a large proportion of the overall weight of medium term followup subgroup analysis. Therefore, the overall effect of medium term subgroup is consistently influenced by this large size study. Furthermore, studies from this subgroup showed a high heterogeneity in terms of time of depression detection and several other characteristics, for example, recruited population and sample size. Paolucci and colleagues [17] evaluated depression within the first nine months after stroke. Both House et al. [10] and Willey et al. [19] assessed depression up to 30 days after stroke, whereas Williams et al. [20] collected information on depression in the first 3 years, excluding those who die within 30 days after stroke. Therefore, it is important to highlight the low degree of comparability between studies. Further studies exploring the influence of duration of followup on the depression mortality after stroke association are probably needed.

Finally, also the HR pooled analysis showed a significant relationship between depression after stroke and mortality. There was a high level of heterogeneity among the included studies in terms of results, recruited population, sample size, and duration of followup. However, the lack of included papers for this analysis did not allow for better exploring the variations of HR through subgroups or sensitivity analyses. It should be noted that the study of Williams and colleagues [20] explored the HR of mortality, but using adjusted measures not suitable for our meta-analysis. The study found a significant HR (HR = 1.13; 95% CI: 1.06–1.21). This result is in line with the overall effect of our meta-analysis and might be taken in consideration when we consider the HR of mortality in subjects suffering from both stroke and depression. 

### 4.2. Strengths and Limitations

To our knowledge, this is the first meta-analysis that systematically synthesizes data from studies comparing the mortality among individuals with and without depression after a stroke. The well-known advantage of a meta-analysis of observational studies is that it allows the synthesis of the results of a large amount of studies, providing findings more robust than those deriving from data of individual studies. 

Observational studies are an important source in epidemiological research, but they are prone to many methodological issues [41, 42]. Therefore, we paid critical attention to the quality of evaluated papers. According to quality assessment, we found methodological issues and potential risk of bias related to selection of sample or depression assessment in some studies. However, the sensitivity analyses did not show significantly different results than the overall pooled analysis. Therefore, lower quality related to poor level of representativeness of selected samples, either lack of reliability or specificity of methods to detect depression, did not seem consistently affecting and influencing the association between depression after stroke and subsequent mortality. However, we need to highlight the low degree of comparability between studies. Studies differ for several important characteristics, other than followup duration, such as study design, source of recruitment, and time of depression assessment.

Our paper included only published studies with sufficient data, excluding conference abstracts because these often cannot give reliable information on patients' characteristics, inclusion criteria, exposure detection, and other relevant issues. Furthermore, hand searching and searching of the grey literature were not conducted, and possibly some relevant studies could not be included. Therefore, we need to consider the risk that an amount of negative or uncertain results remained unpublished or, at least, were not available from databases that we have explored. However, Egger's test showed the lack of risk of publication bias. 

### 4.3. Clinical Perspectives

The nature of the relationship between depression and mortality remains unknown. Depression may affect prognosis and risk of mortality after stroke because stroke patients suffering from depression may be less compliant to treatment. When mental health disorders cooccur with other medical conditions, this cooccurrence tends to reduce adherence to interventions [43, 44].

On the other hand, the relationship may be explained by the fact that depression could be more frequent in people vulnerable to physical disability and a higher stroke severity [45]. In that case, depression may be not an independent factor, but simply a mediator variable for severe physical damage related to a higher likelihood of mortality. 

Emerging line of evidence highlighted that the relationship between depression and stroke and other severe illnesses, for example, myocardial infarction, heart disease, and cancer, is bidirectional and, at least in part, is driven by several biological processes, including immune dysregulation [46]. Depression may lead to dysregulation of immunologic mechanisms, coagulation abnormalities, and vascular endothelial dysfunction, which are associated with an increased risk of cardiovascular disease and mortality [47]. 

Clinicians should regularly assess symptoms of depression in people who report a stroke in their clinical history. Sensitivity and specificity of assessment and screening of depression among people with stroke represent often an important issue. Frequently, a mood disorder remains undetected and, therefore, undertreated [28, 48]. Depressive disorders among patients with anosognosia, neglect, or aprosody, who deny symptoms of depression, may also be underdiagnosed, although symptoms related to specific physical disease, such as changes in appetite or insomnia, may be overestimated by clinicians [26]. 

Equally, treatment of depression is often complicated as people with stroke are often more prone to side effects and interactions among different drugs rather than general population [40], likewise similar comorbidities in mental health disorders [44]. Main data on drugs therapy showed the importance of antidepressant medications, particularly with SSRI, as this may improve not only the life expectancy of poststroke patients but also their quality of life. Trials are limited and focused mainly on antidepressant agents such as Fluoxetine [49, 50], Citalopram [51, 52], and Reboxetine [52, 53]. Systematic reviews found that antidepressants usage may reduce symptoms of depression, but it also pointed out that clinicians should use these drugs with caution in people with persistent depression, as little is known about the risks, especially of seizures, falls, and delirium [54]. Furthermore, there was no clear effect of pharmacological therapy on the prevention of depression after stroke [55].

### 4.4. Conclusions

Despite some limitations, the results of this meta-analysis confirm the potential role of depression on poststroke mortality. Regular screening might help in detecting prevalent cases [48]. Further research is needed in order to clarify the nature of depression poststroke/mortality association and related pathophysiological processes. Secondly, effectiveness of pharmacotherapy and psychotherapy for preventing and treating depression after stroke should be explored. Before any recommendation on their routine use, given the well-known implementation issues of the even more robust guidelines [56], further randomized controlled trials are needed to estimate effectiveness of antidepressants for depression treatment and their potential benefits in terms of life expectancy. 

## Figures and Tables

**Table 1:** Articles suitable for meta-analysis.

Study	Country	Participants	Recruitment	Depression after stroke assessment	Followup	Reported results on mortality
Almeida and Xiao, 2007 [8]	Australia	574 males 55%	Patients with first-ever diagnosis of stroke from January to December 1990	First diagnosis of ICD-9 and ICD-10 depressive disorders recorded during the 24 months following the stroke	10 years	RR: 1.72 (0.98–3.01) HR: 1.26 (0.71–2.23) (depression versus control without mental disorder)

Ellis et al., 2010 [9]	USA	124 males 48%	Participants between 25 and 74 years with stroke diagnosis from NHANES I Epidemiologic Followup Study (NHEFS) interviewed in 1982	Center for Epidemiologic Studies Depression Scale (CES-D) ≥ 16	8 years	Mortality rate (per 1000): 105.1 versus 84.7 (depression versus no depression)

House et al., 2001 [10]	UK	448 males 54%	Patients with definite clinical diagnosis of stroke (not subarachnoid hemorrhage) from a randomized controlled trial	ICD-10 major depression at 1 month after stroke, according to Present State Examination	12 and 24 months	OR at 12 months: 1.3 (0.65–2.7) (major depression) OR at 24 months: 1.7 (0.95–3.0) (major depression)

Jia et al., 2006 [11]	USA	5825 males 98%	Patients with stroke diagnosis between October 2000 and September 2001 from a cohort of veterans, who survived 60 days or more after stroke, and with an index length of stay less than 365 days	Depression (primary or secondary diagnosis) according to ICD-9 codes and antidepressant medication dispensing within 12 months of the index stroke	12 months	Crude death rate: 11.0% versus 12.0% (depression versus no depression)

Jorge et al., 2003 [12]	USA	104 males	Patients between ages 25 and 89 years with acute stroke within the previous 6 months, between June 1991 and June 1997 and from double-blind, placebo-controlled trial	DSM-IV depression due to stroke, with “major depressive-like episode” or “minor depressive disorder,” according to the Present State Examination and Hamilton Depression Rating Scale	9 years	Prevalence of mortality: 25/56 (45%) versus 25/48 (52%) (depression versus no depression)

Kemper et al., 2011 [13]	Germany	977 males 71%	Patients aged 50 years and older with first ischemic stroke in 2005, without previous aphasia, dementia, depression, or nursing care dependency	Diagnosis of depression within the year after stroke, according to ICD-10 codes	12 months after stroke	Adjusted OR: 0.91 (0.55–1.52) (depression versus no depression)

Morris et al., 1993a [14]	Australia	84 males 54%	Patients with stroke undergoing rehabilitation consecutively enrolled from 1986 to 1987 examined approximately two months after stroke (mean 7.6 weeks)	DSM-III major depression approximately 2 weeks after stroke according to Composite International Diagnostic Interview (CIDI) and Montgomery and Asberg Depression Rating Scale (MADRS)	15 months after the initial evaluation (mean 59 weeks)	Prevalence of mortality: 3/13 (23%) versus 1/48 (2%) (major depression versus no depression)

Morris et al., 1993b [15]	USA	91 males: 59%	Patients consecutively admitted to a university hospital stroke unit between 1979 and 1981 with either thromboembolic cerebral infarction or intracerebral hemorrhage	DSM-III major depression 1–3 weeks after stroke according to Present State Examination and Hamilton Depression Rating Scale	10 years	Prevalence of mortality: 26/37 (70%) versus 22/54 (41%) (major depression versus no depression)

Naess et al., 2010 [16]	Norway	771 (376 returning questionnaire) males 60%	Patients with acute stroke consecutively admitted to the Stroke Unit, Haukeland University Hospital, Norway, from February 2006 to November 2008	Hospital Anxiety and Depression Scale (HADS-D) ≥ 11 (at least 6 months after stroke)	Mean followup: 382 days (range 185–756)	HR: 4.4 (*P* = 0.002) (depression versus no depression)

Paolucci et al., 2006 [17]	Italy	1064 males 60%	Patients with ischemic or hemorrhagic stroke (first or subsequent event) confirmed by neuroimaging (CT or MRI), consecutively admitted to one of the study centers between June 2000 and July 2001 (DESTRO study)	Depression within the first 9 months after the stroke according to a Beck Depression Inventory (BDI) ≥ 10	2 years	Prevalence of mortality: 5.48 % versus 4.85% (depression versus no depression)

Ried et al., 2011 [18]	USA	790 males 98%	Patients with a stroke diagnosis between July 2000 and September 2001, from a cohort of veterans	Major depressive disorder or depressive disorder NOS according to ICD-9 codes during the 12 months after stroke	7-year follow-up period (maximum follow-up time: 2465 days)	HR: 1.28 (0.96 to 1.71) (depression versus no depression)

Willey et al., 2010 [19]	USA	340 males 42%	Patients with first-ever ischemic stroke between July 1993 and July 1997, aged >39 years (data deriving from the Northern Manhattan Stroke Study (NOMASS))	First question on the Hamilton Depression Rating Scale regarding their mood in the week after the onset of the stroke (assessment within 30 days of their stroke)	5 years from initial stroke	Adjusted HR: 1.15 (0.76–1.75) (depression versus no depression)

Williams et al., 2004 [20]	USA	51119 males 98%	Patients with a first ischemic stroke from a cohort of veterans who survived beyond 30 days afterward, from October 1990 to September 1998	Diagnosis of depression in the first 3 years after stroke according to ICD-9 codes	3 years after stroke	Adjusted HR: 1.13 (1.06–1.21) (depression versus no depression)
